# Appendiceal Neuroendocrine Tumor Is a Rare Cause of Ectopic Adrenocorticotropic Hormone Syndrome With Cyclic Hypercortisolism: A Case Report and Literature Review

**DOI:** 10.3389/fendo.2022.808199

**Published:** 2022-02-18

**Authors:** Yu Xing Zhao, Wan Lu Ma, Yan Jiang, Guan Nan Zhang, Lin Jie Wang, Feng Ying Gong, Hui Juan Zhu, Lin Lu

**Affiliations:** ^1^ Key Laboratory of Endocrinology of National Health Commission, Department of Endocrinology, Peking Union Medical College Hospital, Chinese Academy of Medical Science and Peking Union Medical College, Beijing, China; ^2^ Department of General Surgery, Peking Union Medical College Hospital, Chinese Academy of Medical Science and Peking Union Medical College, Beijing, China

**Keywords:** Cushing’s syndrome, ectopic ACTH syndrome (EAS), cyclic Cushing’s syndrome, appendiceal neuroendocrine tumor, ^68^Ga-DOTATATE-PET-CT, case report

## Abstract

**Objective:**

Ectopic adrenocorticotropic hormone (ACTH) syndrome (EAS) is a condition of hypercortisolism caused by non-pituitary tumors secreting ACTH. Appendiceal neuroendocrine tumor as a rare cause of ectopic ACTH syndrome was reported scarcely. We aimed to report a patient diagnosed with EAS caused by an appendiceal neuroendocrine tumor and summarized characteristics of these similar cases reported before.

**Case Report and Literature Review:**

We reported a case with Cushing’s syndrome who was misdiagnosed as pituitary ACTH adenoma at first and accepted sella exploration. Serum and urinary cortisol decreased, and symptoms were relieved in the following 4 months after surgery but recurred 6 months after surgery. The abnormal rhythm of plasma cortisol and ACTH presented periodic secretion and seemingly rose significantly after food intake. EAS was diagnosed according to inferior petrosal sinus sampling (IPSS). Appendiceal mass was identified by ^68^Ga-DOTA-Tyr3-octreotate (DOTATATE)-PET-CT and removed. The pathological result was consistent with appendiceal neuroendocrine tumor with ACTH (+). The literature review demonstrated 7 cases diagnosed with EAS caused by appendiceal neuroendocrine tumor with similarities and differences.

**Conclusion:**

The diagnosis of an ectopic ACTH-producing tumor caused by the appendiceal neuroendocrine tumor can be a challenging procedure. Periodic ACTH and cortisol secretion may lead to missed diagnosis and misdiagnosis. IPSS is crucial in the diagnosis of EAS and ^68^Ga-DOTATATE-PET-CT plays an important role in the identification of lesions.

## Introduction

Cushing’s syndrome (CS) is a condition of pathological hypercortisolism, which is classified into adrenocorticotropic hormone (ACTH)-dependent and ACTH-independent causes, in which ectopic ACTH syndrome (EAS) belonging to ACTH-dependent CS is a rare cause, which accounts for 5%–10% of CS (1, 2). The typical EAS is characterized by rapidly progressive clinical figures, including fatigue, severe hypokalemia, myasthenia, and serious infection. Therefore, early diagnosis and removal of responsible lesions are crucial, in order to remit hypercortisolism and prevent fatal complications. However, various difficulties may block proper diagnosis and treatment such as cyclic CS or occult EAS.

The common tumors that contributed to EAS had been published including pulmonary or thymic neuroendocrine neoplasms (NENs), pancreatic neuroendocrine tumors (NETs), pheochromocytomas, and medullary thyroid carcinomas (3). Our center had summarized the clinical spectrum of 88 patients diagnosed with EAS, and it was found that thoracic origins (80.7%) were the most common cause of EAS, comprising pulmonary NETs (43.2%, 38/88), thymic/mediastinal NETs (33%, 29/88), and small cell lung cancer (SCLC) (3.4%, 3/88). Pancreatic NETs were found in 6 patients (6.8%) (4). An appendiceal NET is such as rare cause of EAS that there were only seven cases published until now worldwide (5–9).

Here, we report a case of a 34-year-old woman diagnosed with ectopic CS that was caused by excessive ACTH secretion by the appendiceal NET.

## Case Report

**Figure 1:**
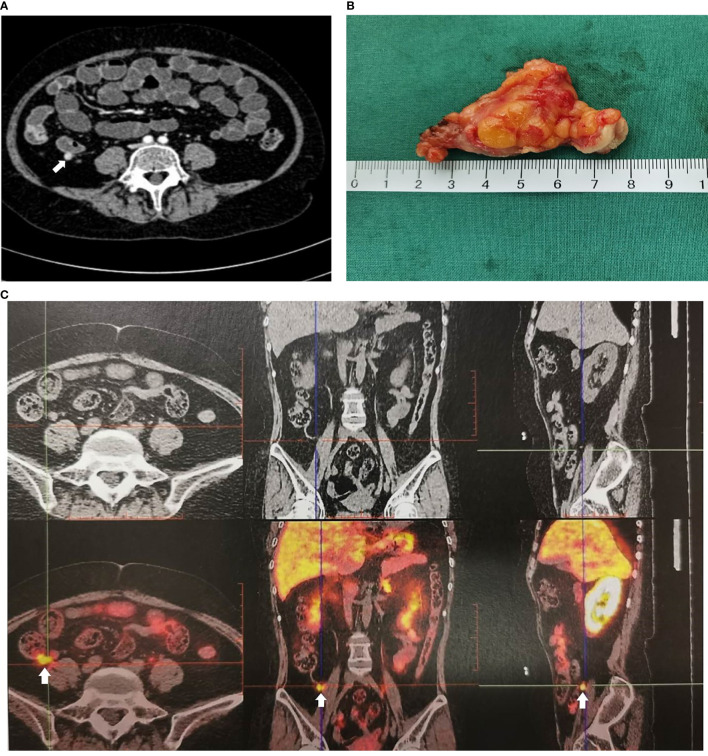
Imaging examination of appendiceal mass. **(A)** Appendiceal mass showed by contrast enhanced CT reconstruction of the small intestine. **(B)** Appendiceal mass. **(C)** Appendiceal mass showed by ^68^Ga-DOTATATE-PET-CT.

A 34-year-old female patient was admitted to our hospital due to hypertension and menstrual disorder. Two years before, she gradually developed a moon face, truncal obesity, acne, and hypertension during pregnancy and menopause after delivery. The level of blood potassium was approximately 2.66~3.64 mmol/L (3.5–5.5). She was diagnosed with Cushing’s disease in a local hospital depending on elevation levels of 24-h urinary free cortisol (UFC) of 1,155.4 μg (12.3–103.5) and ACTH of 18.2 pg/ml (0–46) (**Table 1**) and suspicious pituitary microadenoma in MRI initially. Consequently, a neurosurgical sella exploration was performed. Postoperative pathology did not conform to ACTH pituitary tumors. Nevertheless, the patient presented peeling and improvement of acne and hypokalemia with reduction of ACTH (13.1 pg/ml), plasma cortisol (8.3 μg/dl), and 24-h UFC (104.4 μg) 4 months after surgery (**Table 1**). The remission of hypercortisolemia was considered at that time. However, 6 months later, severe fatigue, hypokalemia, and hyperglycemia reappeared with an elevation of 24-h UFC (358.2 μg) and plasma cortisol of 720 nmol/L (133–537) (**Table 1**). The patient came to our clinic for further treatment. She has no special past or family history. Physical examination showed typical cushingoid features including moon facies, supraclavicular fatty pad, buffalo hump, skin atrophy, wide purple striae, purpura, and hirsutism. Further examination found hypokalemia, hypertension, osteoporosis (multiple rib fractures), and urolithiasis. Endocrine hormone-related examination revealed elevated levels of 24-h UFC (615.7~837.1 μg). The level of 8 a.m. serum cortisol fluctuated between 21.8 and 25.2 μg/dl, and ACTH was between 17.7 and 19.8 pg/ml (**Table 1**). Further examination suggested an abnormal rhythm of plasma cortisol and ACTH, which presented periodic secretion in 4-h cycles and seemingly rose significantly after meals (**Table 2**). A low-dose dexamethasone suppression test (LDDST; 2 mg of dexamethasone test) confirmed the diagnosis of hypercortisolism. A high-dose dexamethasone suppression test (HDDST; 8 mg of dexamethasone test) was not greater than 50% suppression of 24-h UFC from the basal value. Inferior petrosal sinus sampling (IPSS) revealed a central/peripheral ACTH ratio of less than 2 and less than 3 with desmopressin injection. The peripheral desmopressin stimulation test is shown in **Table 2**, which suggested a positive response to desmopressin. Diagnosis of ectopic ACTH syndrome with the cyclic secretion of ACTH (cyclic CS) was possible. In order to localize the source of ACTH secretion, the following were completed with no hint: octreotide scan; contrast-enhanced CT of the chest, abdomen, and pelvis; and ^18^F-fluorodeoxyglucose (FDG)–positron emission tomography (PET)–CT. Finally, ^68^Ga-DOTA-Tyr3-octreotate (DOTATATE)-PET-CT was done, and an abnormal increase in radioactivity uptake was observed in the appendix, with a SUVmax of 17.1. Contrast-enhanced CT reconstruction of the small intestine also suggested a mass in the lumen near the blind end of the appendix with obvious enhancement (**Figure 1**). The patient had no history of abdominal pain, changes in bowel habits, or rectal bleeding; neither did she have any complaints of flushing or palpitation. Combined with the above examination results, it was considered that the responsible lesion was appendiceal mass, possibly an appendiceal NET. So a laparoscopic appendectomy was performed, and a mass measuring 1.4 cm × 0.6 cm × 0.5 cm was found in the blind side of the appendix (**Figure 1B**). Pathology examination revealed appendiceal NET (G2, mitotic 4/10HPF) invading the muscle layer of the appendix and the surrounding appendiceal tissue, and abnormality was not observed at the incisor margin. *Immunohistochemistry*: CgA (+), Ki-67 (index 3%), SYN (+), TTF-1 (−), CD56 (+), P53 (wild type), ATRX (+), SSTR2 (+), and ACTH (+). Serum cortisol dropped to 2.5 μg/dl, and ACTH decreased below 5 pg/ml the first day after the operation, suggesting remission of ectopic CS. Hydrocortisone replacement was given and gradually tapered down. Hypokalemia was treated and menstruation resumed, with weight loss and peeling 3 months after surgery.

**Table 1:** Laboratory results of the patient.

Time	ACTH (pg/ml) (RR: 0–46)			Serum cortisol (μg/dl) (RR: 4–22.3)			24-h UFC (μg)(RR: 12.3–103.5)
	8 a.m.	4 p.m.	Midnight	8 a.m.	4 p.m.	Midnight	
Before surgery	18.2	57.36	56.55	346.4^#^	764.2^#^	719.9^#^	455.7–1115.4
Postoperative day 1	25.61	68.82		684.5^#^	1158^#^		
4 months after surgery	13.1			8.3			104.4
10 months after surgery	10.4	13.6	13.1	472^#^	717.5^#^	720.1^#^	
15 months after surgery	25	91.8	/	24.1	52.15	16.34	615.7
15 months after surgery	19.8	65.1	/	25.2	53.88	/	

The rhythm of serum cortisol and ACTH in this patient.

ACTH, adrenocorticotropic hormone; UFC, urinary free cortisol.

^#^The unit of serum cortisol is nmol/L; reference range (RR) is 133~537.

**Table 2:** Laboratory results of the patient.

Monitoring of serum cortisol for 18 h (μg/dl)														
6 a.m.	8 a.m.*		10 a.m.		Noon*	2 p.m.		4 p.m.	6 p.m.*		8 p.m.		10 p.m.	Midnight
14.6	40.1		45.6		41	31.3		26.4	42.5		34.8		22.1	16.34
Peripheral desmopressin stimulation test														
			0 min		15 min	30 min		45 min	60 min		90 min		120 min	
ACTH (pg/ml)			19.4		23.7	17.9		15.7	13.5		11.9		36.0	
Serum cortisol (μg/dl)			10.3		21.2	21.9		18.2	16.6		14.8		24.4	
Inferior petrosal sinus sampling and desmopressin test (ACTH: pg/ml)														
Time		Periphery		Left inferior petrosal sinus			Right inferior petrosal sinus			Left internal carotid		Right internal carotid		Central/peripheral
0 min		16.5		19.4			20.5			19.8		20.1		1.24
3 min		27.2		41.6			45.5							1.67
5 min		32.0		48.1			48.8							1.53
10 min		42.7		57.2			57.2							1.34

ACTH, adrenocorticotropic hormone.

^*^The patient just finished food intake.

## Literature Review of Cushing’s Syndrome Caused by Appendiceal Neuroendocrine Tumor

CS caused by appendiceal NETs is extremely rare. To our knowledge, only seven other cases have been reported until now. Here, we summarized and analyzed the characteristic features of these cases shown in **Table 3**. Interestingly, except for a 15-year-old girl, all of these reported cases were young women in their 20s~30s with typical cushingoid features and weight gain. Five patients showed an appearance of hyperandrogenemia including hirsutism or acne, but only one patient presented abdominal symptoms such as constipation and chronic abdominal pain. Hypertension, diabetes, and severe osteoporosis also have been found. Diagnosis of CS was certified by elevated 24-h UFC and serum cortisol, as well as LDDST. Levels of ACTH measured in five of these patients were all greater than 20 pg/ml, supporting ACTH-dependent CS. Successful IPSS was done in three patients who presented no gradient, supporting ectopic ACTH syndrome. It is difficult to find ectopic lesions, especially in the early disease course when PET-CT was not available. In two cases, the appendiceal lesions were found during exploratory laparotomy for the proposed adrenal resection. In the other four patients, ^18^F-FDG-PET-CT, DOPA-PET-CT, ^68^Ga-DOTATE-PET-CT, and ^99m^Tc HYNIC-TATE-PET-CT were used to identify the responsible lesion in the appendix. All seven patients underwent appendectomies, and four of them underwent hemicolectomy, which resulted in remission of CS. Pathological results were all consistent with appendiceal NETs, and four of them had positive ACTH staining. Immunostaining for ACTH and pro-opiomelanocortin in case 6 was negative, which did not support diagnosis and may suggest that the tumor either was unrelated to the EAS or represents dedifferentiation of the original tumor. Two of seven patients presented periodic cortisol secretion.

**Table 3:** Characteristics of cases with IGSF1 deficiency from published literature.

	Case 1	Case 2	Case 3	Case 4	Case 5	Case 6	Case 7
Author	Timothy Miller (5)	H. Dobnig (6)	David Beddy (7)	N. Perakakis (8)	Chakra Diwaker (9)	Ashley B. Grossman (10)	Elżbieta Moszczyńska (11)
Year published	1971	1996	2010	2011	2019	1986	2021
Nation	USA	Germany	USA	Germany	India	UK	Poland
Sex	Female	Female	Female	Female	Female	Female	Female
Age at onset	35	33	23	31	22	24	15
Age at surgery of appendectomy	36	/	23	32	/	44	17
Weight gain	+	+	+	+	/	+	+
Cushingoid features	+	+	+	+	/	+	+
Menstrual disorder	+	+	−	+	/	+	+
Hirsutism or acne	−	+	+	+	/	+	+
Hypertension	+	−	+	−	/	−	−
Diabetes	−	−	−	+	/	−	−
Hypokalemia	+	−	−	−	/	+	+
Osteoporosis	+	+	−	−	/	−	−
Urolithiasis	−	−	−	−	/	−	−
Others	Emotional instability	Swelling	Weakness	Constipation, chronic abdominal pain	/	Edema	Sinus bradycardia
24-h UFC	/	/	1,663 μg (3.5–45)	Elevated	/	/	/
Serum cortisol	10 (5–20)	629 nmol/L (138–689)	31 μg/dl (7–25)	73 μg/dl	/	>2,000 nmol/l	47.1 μg/dl
ACTH	/	6.4 pmol/L (2.2–11)	/	182 pg/ml	8 pmol/L (0–10)	48~204 pg/l	182
Periodic secretion	Not mention	Not mention	Not mention	Not mention	Not mention	+	+ (every 1–2 months)
LDDST	Not suppressed	/	Not suppressed	Not suppressed	Not suppressed	Not suppressed	/
HDDST			+	Suppressed more than 50%	/	Suppressed less than 50%	Suppressed less than 50%
CRH testing	/	No response	/	No response	/	No response	Contradictory
IPSS	/	0.8	No gradient	Not success	1.3:1	Not success	/
Pituitary MRI		+	−	−	−	−	+
Abdominal CT	/	−	2 cm mass medial to the cecum	−	A small lesion from the appendix or cecal mesentery	−	2.4-cm abnormal density in the appendix region
Octreotide scan	/	/	/	−	/	−	/
Fu-PET-CT	/	/	2-cm mass medial to the cecum	−	/	/	/
DOPA-PET-CT/^99m^Tc HYNIC-TATE	/	/	/	1.8 × 1.1 cm mass in the terminal ileum	1.0 × 1.2 × 1.9 cm (SUVmax, 9.5) in the appendix	/	Increased radiotracer uptake in the appendix
Colonoscopy	/	−	Submucosal mass in the cecum	−	/	−	/
Treatment	Abdominal exploration + appendectomy + adrenalectomy	Appendectomy + right-sided hemicolectomy and lymphadenectomy	Right hemicolectomy	Appendectomy 1 year later operated right hemicolectomy	Laparoscopic appendectomy	Adrenolytic therapy trans-sphenoidal surgery Bilateral adrenalectomy appendectomy with en bloc resection of the adherent mesentery	Laparoscopic appendectomy Right-sided hemicolectomy
Pathology examination	Appendiceal carcinoid without invasion	Infiltrating carcinoid tumor	ACTH-producing carcinoid tumor of the appendix	Appendiceal neuroendocrine tumor infiltration of the submucosal and subserosa of the pericolic fat and vascular invasion (G1)	ACTH-secreting appendicular carcinoid	Carcinoid tumor with metastases to mesenteric and local nodes	Moderately differentiated NET G2, with metastasis of peritoneum, mesentery, greater omentum, lymph nodes
Histological examination	Positive argentaffinoma reaction	ACTH (+) Neuron-specific enolase (+) Chromogranin (+)	Chromogranin (+) Synaptophysin (+) ACTH (+)	ACTH (+) Chromogranin (+)	/	ACTH (−) Pro-opiomelanocortin (−)	Chromogranin A (+) Synaptophysin (+)
Follow-up	Remission	Remission	Remission	Remission	Remission	/	Remission Recurrence in 7 years

UFC, urinary free cortisol; ACTH, adrenocorticotropic hormone; LDDST, low-dose dexamethasone suppression test; HDDST, high-dose dexamethasone suppression test; CRH, corticotropin-releasing hormone; IPSS, inferior petrosal sinus sampling.

+, the patient had this symptom; −, patient did not have this symptom; /, this result was not mentioned in the report.

## Discussion

Appendiceal NETs represent the most common tumor of the appendix, found in 0.2%–0.7% of all appendectomies (12) and accounting for 2%–5% gastrointestinal NETs (13), commonly being identified incidentally during appendectomy performed for appendicitis. Appendiceal NETS are more common in women and are mostly submucosal at the tip of the appendix. They are less likely to cause obstruction and are mostly asymptomatic (13). NETs originate from neuroendocrine cells, secreting different substances such as somatostatin, gastrin, and ACTH. Excess amounts of these substances can lead to various clinical presentations; for instance, excess ACTH secretion induced CS.

Based on this case report and literature review, the diagnosis of CS caused by appendiceal NETs is challenging; even some patients were misdiagnosed to have pituitary ACTH microadenoma, and they underwent pituitary exploration surgery before the discovery of the appendiceal tumor. The main reasons for the difficulty in diagnosis were as follows. First of all, specific clinical manifestations were lacking. Cushing-like appearance, hypertension, diabetes, hypokalemia, and osteoporosis are still the main clinical manifestations. Unexpectedly, abdominal symptoms caused by appendiceal masses were not prominent, and only one patient has relevant symptoms, which was consistent with previous reports on asymptomatic appendiceal NETs (14).

Secondly, it is always challenging to distinguish between Cushing’s disease and ectopic ACTH-dependent CS by tests. IPSS has long been the gold standard to reliably exclude ectopic ACTH production but should preferably be performed in a specialized center because of potential patient risk. If IPSS was not allowed, a combination of three or four non­invasive approach tests was recommended, specifically corticotropin-releasing hormone (CRH) and desmopressin stimulation plus MRI, followed by whole-body CT if the diagnosis is equivocal (15). Unfortunately, the hospital where this patient was first admitted did not have the capacity to implement IPSS and CRH or desmopressin stimulation tests. Misdiagnosis led to inappropriate treatment options and prolonged illness. In addition, it is difficult to locate space-occupying lesions of the gastrointestinal tract by conventional imaging such as CT. It is gratifying that more and more highly specific imaging techniques are being used in the clinic. Somatostatin receptor scintigraphy (SRS), ^18^F-FDG, or ^68^Ga-DOTATATE-PET/CT was considered if CT or MRI did not show typical findings of ectopic tumors (4). Compared to the other two nuclear functional imaging techniques, ^68^Ga-DOTATATE-PET-CT had a higher affinity for the SSTR2 receptor and higher resolution to illustrate anatomical details, helping in the successful detection and accurate localization of small tumors characterized by SSTR (16–18). Taweesak et al. found that ^68^Ga-DOTATATE identified the primary ECS in 11/17 (65%) of previously occult tumors (19). The case in our report failed to identify the responsible lesions by CT, octreotide imaging, and ^18^F-FDG-PET-CT. Finally, ^68^Ga-DOTATATE-PET/CT successfully located the lesion in the appendix.

Periodic cortisol secretion also contributed to another difficulty in this case. Diagnosis of cyclic CS is based on at least three periods of confirmed hypercortisolemia interspersed by two periods of normocortisolemia (20). Although these criteria apply to most patients, they might be hard to fulfill in others, particularly if due to the long intercyclic phase or intermittent hypercortisolism. The presence of only two peaks and one trough of hypercortisolism, in this case, did not conform to the diagnostic criteria. But given the overall course of the disease, this patient is highly likely to be diagnosed with cyclic Cushing’s syndrome. The trough of cortisol production occurred just after sella exploration, giving the false appearance of recovery, which led to an incorrect diagnosis of pituitary ACTH adenoma. Meanwhile, the case showed great increasing levels of serum cortisol and ACTH at 4 p.m. compared to normal levels at 8 a.m., which may result in missed diagnosis of ACTH-dependent hypercortisolism if the blood sample at 4 p.m. was not collected. To further explore the rhythm of cortisol secretion in this patient, levels of serum cortisol were monitored every 2 h (from 8 a.m. to 12 p.m.) and suggested another shorter periodic secretion. Interestingly, most of the peaks seemed to appear after food intake, which had not been reported in previous case reports. A review of 65 reported cases demonstrated that cyclic CS originated in 26% from an ectopic ACTH-producing tumor, which included thymic carcinoid, bronchial carcinoid, pancreatic carcinoid, renal carcinoid, gastric carcinoid, epithelial thymoma, pheochromocytoma, and 2 occult cases. Cycle length ranged from 4 to 180 days (20). In a review of 7 cases of EAS caused by appendiceal carcinoids reported before, two of them showed periodic hormone secretion, but neither of these secretion cycles was as short and related to food consumption as in this case. We speculated that food residues flowing through the intestine and intestinal peristalsis after food intake might stimulate ACTH secretion of appendiceal NET. But it is a pity that we did not extend it for a longer time to confirm the association with food intake to distinguish with a variable secretion of cortisol.

The criteria of the desmopressin test associated with the best compromise between sensitivity and specificity were a relative cortisol increase >18% and ACTH increase >33% for the desmopressin test with 83% sensitivity and 81% specificity for the diagnosis of Cushing’s disease (21). This patient presented a positive response to desmopressin, which confirm Cushing’s disease, not EAS. However, it has been shown that some cases of EAS expressed the V2 receptor and responded to desmopressin (22). Periodic cortisol secretion may also disturb the result, which can explain the result of this patient.

For most T1 appendiceal tumors confined to the appendix, simple appendectomy is sufficient because of infrequent metastasis. In those with tumors larger than 2 cm, right hemicolectomy is recommended. Controversy exists regarding the management of appendiceal NETs measuring less than 2 cm with more aggressive histologic features. It has been suggested that the presence of lymph node metastases in appendiceal NETs smaller than 2 cm may lead to more aggressive management of appendiceal NETs with adverse prognostic factors (lymphovascular or mesangial invasion or atypical histological features). For these tumors, right hemicolectomy is recommended (23).

In conclusion, eight cases of an EAS due to appendiceal NETs have been reported until now. These cases demonstrate that the diagnosis of an ectopic ACTH-producing tumor can be a challenging procedure, which demands a systemic multidisciplinary approach, regular follow-ups, and the use of various novel imaging techniques. Periodic hormone secretion may be confusing, so there should be careful attention to screening and examination. For occult EAS, gastrointestinal NETs should not be ignored.

## Data Availability Statement

The original contributions presented in the study are included in the article/supplementary material. Further inquiries can be directed to the corresponding author.

## Ethics Statement

The studies involving human participants were reviewed and approved by the Ethics Committee of Peking Union Medical College Hospital. Written informed consent for participation was not required for this study in accordance with the national legislation and the institutional requirements. Written informed consent was obtained from the individual(s) for the publication of any potentially identifiable images or data included in this article.

## Author Contributions

YZ contributed to the conception and design of the study, diagnosis and treatment of the patient, literature review, and drafting of the manuscript. WM contributed to the diagnosis and treatment of the patient. YJ contributed to the diagnosis and treatment of the patient. GZ contributed to the treatment of the patient. LW critically revised the manuscript for important intellectual content. FG critically revised the manuscript for important intellectual content. HZ critically revised the manuscript for important intellectual content. LL contributed to the conception and design of the study, and diagnosis and treatment of the patient, and critically revised the manuscript for important intellectual content. All authors listed have made a substantial, direct, and intellectual contribution to the work and approved it for publication.

## Conflict of Interest

The authors declare that the research was conducted in the absence of any commercial or financial relationships that could be construed as a potential conflict of interest.

## Publisher’s Note

All claims expressed in this article are solely those of the authors and do not necessarily represent those of their affiliated organizations, or those of the publisher, the editors and the reviewers. Any product that may be evaluated in this article, or claim that may be made by its manufacturer, is not guaranteed or endorsed by the publisher.
